# Current Therapy of Chronic Viral Hepatitis B, C and D

**DOI:** 10.3390/jpm13060964

**Published:** 2023-06-07

**Authors:** Jörg F. Schlaak

**Affiliations:** Department of Internal Medicine, Ameos Hospital Oberhausen, Wilhelmstr. 34, 46145 Oberhausen, Germany; jfs63@web.de

**Keywords:** chronic hepatitis B, chronic hepatitis C, chronic hepatitis D, antiviral therapy, liver cirrhosis, DAA therapy, functional cure, HBsAg loss

## Abstract

The majority of chronic viral hepatitis cases are induced via infection with the hepatitis B virus (HBV), hepatitis C virus (HCV), or hepatitis D virus (HDV). These patients are at increased risk for progressive liver disease leading to cirrhosis as well as hepatocellular carcinoma (HCC). HBV infection is well controlled by the currently available nucleosides as well as nucleotides, and the development of cirrhosis can be prevented. Additionally, it has been shown that HBV-induced liver fibrosis can regress during successful antiviral treatment; however, a “functional cure”, i.e., loss of HBsAg, is a rare event when these drugs are used. Therefore, novel therapeutic strategies are aiming at the selective suppression of HBsAg levels in combination with immunostimulation. The development of directly acting antivirals (DAAs) has revolutionized HCV therapy, as almost all patients can be cured via this treatment. Additionally, DAA therapy has few, if any, side effects, and is generally well tolerated by patients. HDV remains the most challenging type of chronic viral hepatitis. Although novel therapeutic options have recently been approved, response rates are still less favorable compared to HBV and HCV. This review discusses current and future options for the treatment of chronic HBV, HCV, and HDV infection.

## 1. Introduction

Chronic viral hepatitis induced via infection with the hepatitis B virus (HBV), hepatitis C virus (HCV), or hepatitis D virus (HDV) is still a major cause for morbidity and mortality worldwide; HBV and HCV are among the 10 major causes of global mortality [1]. It is estimated that 300 million people are chronically infected with HBV, while 58 million are infected with HCV [2] and 20 million are infected with HDV [1,2]. These patients are at increased risk for progressive liver disease leading to cirrhosis as well as hepatocellular carcinoma (HCC).

*HBV.* In general, chronic HBV infection is well controlled by the currently available drugs, and the development of cirrhosis can be prevented. Additionally, it has been shown that HBV-induced liver fibrosis can regress during successful antiviral treatment [3,4]. This requires the careful selection of an antiviral drug (low resistance rate, etc.) and the adequate drug adherence of a patient. In some HBeAg (HBV envelope antigen)-positive patients, antiviral therapy can cause seroconversion into anti-HBe. The elimination of HBsAg (HBV surface antigen), with or without seroconversion into anti-HBs, however, occurs in only a small proportion of patients. Over a period of 5 years of therapy, only 10% of patients may experience HBsAg loss and seroconversion. This so-called “functional healing”, however, is the goal of future treatment strategies against HBV as it leads to a significantly reduced induced risk of HCC and other complications of liver fibrosis.

*HCV.* Similarly to HBV, infection with HCV can lead to serious and potentially life-threatening complications, such as liver cirrhosis or HCC. In the past it was treated with type I interferons (IFNs), which was complicated by numerous side effects and was only effective in a small proportion of patients, largely depending upon the HCV genotype and underlying stage of liver disease. The development of directly acting antivirals (DAAs) has revolutionized HCV therapy, as almost all patients can be cured via this treatment independent of genotype, fibrosis stage, and other risk factors. In addition, DAA therapy has few, if any, side effects, and is generally well tolerated by patients.

*HDV.* HDV remains the most challenging type of chronic viral hepatitis when therapy options are considered [5]. An HDV prevalence of up to 1% of the world population has been suggested, although reliable data on the global HDV prevalence are still missing [6]. It can be assumed that HDV prevalence is even higher in different risk groups, such as patients infected with human immune deficiency virus (HIV), where it is a major cause of liver-related morbidity [7]. Early studies have demonstrated that HDV coinfection leads to liver cirrhosis, liver decompensation, and HCC in a significant proportion of patients [8,9], which could be confirmed in more recent monocentric series [10,11]. Although novel therapeutic options have recently been approved, response rates are still less favorable compared to those seen in the treatment of chronic HBV and HCV infection.

## 2. Current Therapy of Chronic Hepatitis B

### 2.1. Indication for Antiviral Therapy

The suppression of viral load as well as normalization of liver function tests define the indication for the antiviral therapy of chronic HBV infection. Treatment is clearly indicated when transaminases are elevated and the viral load exceeds 2000 IU/mL. Other causes of liver disease, such as nonalcoholic steatohepatitis (NASH), alcohol abuse, etc., or a coinfection with HDV should be excluded, however. Another clear indication for therapy is given in HBV patients with advanced liver disease (F3 fibrosis or liver cirrhosis). Here, treatment should be initiated in all individuals that tested positive for HBV DNA via PCR regardless of transaminase levels or viral load [12,13,14]. This is also true for patients with HBV-related HCC and positive HBV PCR, as the risk of tumor recurrence or progress can be reduced [15,16,17]. Further therapy indications include a reduction in maternal transmission in pregnancy, professional (e.g., medical staff) or social reasons to reduce the risk of transmission, extrahepatic manifestations of HBV infection, and the prevention of HBV reactivation via immunosuppression.

**Figure 1:**
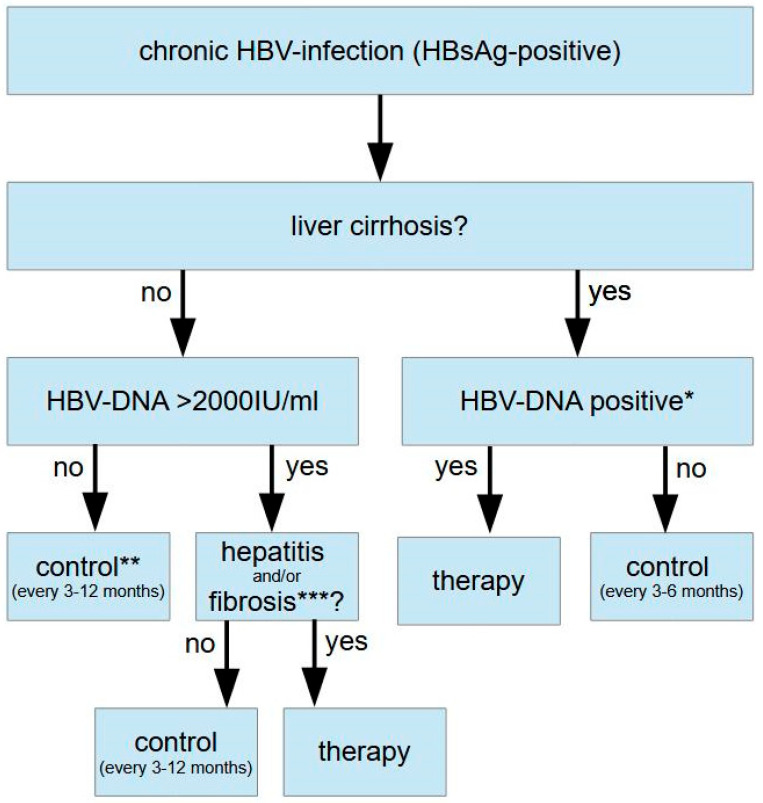
Algorithm for therapy indication in chronic HBV infection (modified from [18]). * Sensitive assay (<12 IU/mL), ** therapy can be indicated for other reasons (prophylaxis, extrahepatic manifestations, reduction in transmission, and HCC risk), and *** histology ≥ F2 fibrosis (Desmet).

The constellation that was earlier described as being an “asymptomatic HBsAg carrier” (normal transaminase levels and an HBV viral load < 2000 IU/mL) is usually no indication for therapy as the risk of liver disease progression and HCC development risk, as well as infectivity, are very low in these patients. The indication for therapy in patients with normal transaminase levels and a very high viral load (earlier named as the “immunotolerant stage”) is still controversial. It has been suggested that antiviral therapy should be initiated in patients >30 years or in individuals with transaminases in the upper range of normal [12] (Figure 1).

### 2.2. Therapeutic Options for the Treatment of Chronic Hepatitis B Infection

Currently, there are two therapeutic strategies available for the treatment of chronic HBV infection: pegylated interferon alpha (Peg-IFN) and nucleoside or nucleotide analogues (NUCs). Peg-IFN is given once a week subcutaneously for 48 weeks and leads to long-term clinical responses (reduction in viral load and normalization of liver function tests) in about one-third of patients. Due to the relatively low response rates that are accompanied by relevant side effects, this therapy regimen is used in only a few patients. Most patients are treated with NUCs such as tenofovir (TDF) or entecavir (ETV), as this is well tolerated and leads to the reliable suppression of viral load as well as the normalization of transaminases. The downside of this approach is that a viral rebound may occur once NUC therapy has been stopped; thus, NUCs have to be given permanently.

*a. PEG-IFN.* IFN treatment may be considered in the presence of favorable baseline predictors, such as the absence of a high viral load (e.g., <1 million IU/mL), significantly increased transaminases, and HBV genotype A [12,13]. In Asian patients, genotype B/C age < 40 yrs., female gender, alanine aminotransferases (ALT) > 4× ULN, HBsAg levels < 25,000 U/mL and HBV DNA < 6 logIU/mL are predictive for a favorable response to PEG-IFN therapy [19]. Contraindications of PEG-IFN (e.g., previous psychiatric illnesses, Child cirrhosis stage B/C, autoimmune diseases, etc.) should be excluded before treatment is initiated. Successful IFN therapy is associated with a drop in HBsAg serum levels at therapy week 12 of more than 20% and below 20,000 IU/mL [18]. If this is not achieved, Peg-IFN therapy should be stopped and the patient should be switched to an NUC-based regimen. A de novo combination therapy with NUCs, an “add-on” of PEG-IFN to NUC therapy, or a switch from NUC- to PEG-IFN treatment is not recommended [18].

*b. NUCs*. Today, the most commonly used NUCs are tenofovir disoproxil (TDF; 245 mg/day) and entecavir (ETV; 0.5–1 mg/day). Lamivudine (LAM), adefovir (ADF), and telbivudine (TVD) are also licensed but are used less often, as high resistance rates have been observed. TDF and ETV are well tolerated, and resistance is only rarely seen. In the large majority of patients they lead to the suppression of the HBV viral load below the limit of detection and to the normalization of transaminases. If this is not achieved, a lack of compliance or therapy adherence of the patient should be considered. Long-term side effects include a slowly progressive reduction in bone density and the deterioration of renal function. Both side effects are more common under TDF as compared to ETV therapy. Therefore, a TDF analogue (tenofovir alafenamid (TAF)) has been developed with higher liver specificity that can be given at a lower dose (25 mg/day), therefore with fewer side effects [20,21]. It has been shown that long-term (5 years and more [22]) NUC therapy can lead to the regression of fibrosis or cirrhosis and may reduce the risk of HCC development. It is still a matter of debate, however, if TDF has stronger effects on the risk of HCC development than ETV [23,24,25].

*c. Duration of NUC therapy.* In (wild-type) HBeAg-positive patients, NUC therapy can be terminated 12 months after seroconversion into anti-HBe, with persistently negative HBV DNA thereafter. In (mutant) HBeAg-negative patients with detectable HBV DNA before therapy, the only definitive end point of NUC therapy is the loss of HBsAg with or without the detection of anti-HB antibodies. In patients with liver cirrhosis, a more cautious approach should be taken as it is recommended to prolong NUC therapy until anti-HB antibodies can be detected. This, however, is a rare event. Therefore, several studies have addressed the question as to whether it is possible to stop NUC therapy in the absence of these clear end points [26,27,28]. The current German and European guidelines consider ending therapy if HBV DNA has been undetectable for at least 3 years, advanced fibrosis is absent, and a close follow-up is guaranteed [12,18]. Further criteria include low HBsAg levels (<100 IU/mL) that have been shown to predict HBsAg loss in Asian patients [29]. Individual studies have identified further parameters, such as a low viral load before therapy (<200,000 IU/mL), low ALT, age < 40 yrs., female gender, and the absence of liver cirrhosis as favorable predictive parameters [30,31]. A relapse usually occurs 1–12 months after the cessation of NUC therapy. Therefore, liver function tests and HBV DNA should be controlled every 4 weeks in the first 6 months and every 12 weeks thereafter. In the case of a relapse, a rise in HBV DNA is initially observed, followed by an elevation in ALT elevation [30,31].

*d. HBV and pregnancy.* In rare cases, the activation of an HBV infection has been observed during pregnancy that can also lead to acute liver failure. In most patients, however, the attenuation of inflammatory activity is seen. After pregnancy, ALT flares can occur in the first 3–6 months after birth. Therefore, ALT and HBV DNA levels should be controlled in HBsAg-positive pregnant women every 3 months until 6 months after birth [18]. In the case of a patient becoming pregnant during antiviral treatment with an NUC or PEG-IFN, therapy with LAM, TVD, and TDF can be continued while PEG-IFN should be stopped, and ETV or ADF should be switched to TDF [18]. In therapy, naïve women initiation of antiviral treatment should be considered when active hepatitis has been diagnosed or HBV DNA > 200,000 IU/mL has been detected. It has been demonstrated that a high viral load increases the vertical mother-to-child transmission of HBV by up to 32%, while this risk is minimized when HBV DNA is suppressed < 200,000 IU/mL [32]. Therefore, antiviral treatment should not be initiated when HBV DNA is below this level. De novo antiviral treatment should be started as early as possible after the first trimester, preferably using TDF, and the patient should be informed about the possible risks and benefits [33]. NUC therapy can be stopped after birth in the absence of a medical indication (e.g., inactive hepatitis) or to prevent the transfer of toxic metabolites during breast feeding; however, it has been demonstrated that breast feeding is safe when TDF is used as an antiviral agent [34,35].

*e. Prevention of HBV reactivation during immunosuppression.* The reactivation of an inactive HBV infection is a potentially life-threatening complication of chemotherapy or immunosuppression. Therefore, HBsAg and anti-HBc should be tested before the initiation of chemotherapy or an immunosuppressive treatment [12,36,37]. In HBsAg-positive carriers, the reactivation of active hepatitis occurs in 15–50% patients after the start of chemotherapy, while this rate reaches 75% after bone marrow transplantation and fulminant hepatitis; fatal outcomes have also occurred. In HBsAg-negative/anti-HBc-positive individuals, the reactivation rate can reach 10%. As a result, it is recommended that HBsAg-positive patients with a high (>10%) risk of reactivation *must* be treated with NUCs, while patients with a moderate (1–10%) risk *should* be treated with NUCs. HBsAg-positive patients with a low (<1%) risk of reactivation should be controlled every 8 weeks (Table 1). As mentioned, the reactivation risk in HBsAg-negative/anti-HBc-positive patients is much lower. Here, only patients with a high risk should be treated (Table 2). The risk of reactivation depends on the type of drug or immunosuppression used (Table 1 and Table 2) [18]. For prophylaxis and preemptive therapy, highly potent NUCs (TDF and ETV) should be used [38,39] for 6–12 months [12,36]. After B-cell-depleting chemotherapy and high-risk constellations, prophylactic NUC therapy should be given for 18 months [40].

### 2.3. Future Options in the Treatment of Chronic Hepatitis B Infection

While viral replication can be controlled and the progression of liver disease can be prevented in most patients that are treated with the currently licensed antiviral drugs against HBV, the long-term control of an infection (“functional cure”) is rarely seen, as HBsAg levels remain mostly elevated despite effective antiviral therapy. It has been demonstrated that HBsAg itself may suppress innate immune responses against HBV in particular, and thus promote the chronicity of an infection [41,42,43,44]. Therefore, several approaches are being developed that are aiming at HBsAg elimination. These can be divided into two groups that are acting directly on the virus (DAA) or that are aiming to improve the antiviral innate and/or adaptive immune response (IAS), respectively (Table 3, from [45]).

*a. Directly acting antivirals (DAAs).* Bulevirtide (BLV) is an inhibitor of HBV entry and targets NTCP (sodium taurocholate cotransporting polypeptide), which functions as a receptor for HBV into a host cell. It is already licensed for the treatment of HBV/HDV coinfection (see below). Here, it has been shown to lead to HBsAg elimination in some cases when combined with PEG-IFN.

Capsid assembly modulators (CAMs) lead to the reduced formation of cccDNA and the introduction of HBV DNA into the nucleus by inhibiting the assembly of the HBV core protein. In clinical studies this has led to the suppression of HBV DNA without affecting HBsAg levels [46].

Very promising clinical results have been generated with HBsAg secretion inhibitors, such as REP-2139 or REP-2165, which are nucleic acid polymers (NAPs). In combination with PEG-IFN, they have demonstrated a high rate of a functional cure (e.g., sustained suppression of HBsAg after therapy) [47]. During therapy, ALT flares have been observed, suggesting the activation of the immune system.

Several small interfering RNAs (siRNAs) and antisense oligonucleotides (Aos) are currently in clinical development that are targeting the production of HbsAg and other viral proteins. In some studies a marked reduction in HbsAg levels was observed [48].

The data so far suggest that a combination of DAAs that lower HbsAg levels with IASs, such as PEG-IFN, may lead to a functional cure in a substantial proportion of patients. It will be of interest to see whether a combination of several DAAs may further enhance this effect.

*b. Immune-activating strategies (IASs).* Our own in vitro and in vivo data have suggested that the activation of the innate immune system may efficiently suppress HBV replication, while high levels of HBsAg may attenuate this immune activation [41,42,43,44]. Human data also suggest that HCV, which activates the innate immune system, can suppress HBV replication in HCV/HBV-coinfected patients. Therefore, toll-like receptor (TLR) agonists were developed to activate the innate immune system. Here, GS-9688, which activates TLR8, has shown some promising first clinical results in human studies and animal models [49,50].

Therapeutic vaccination may represent another approach that is aimed at activating the adaptive immune system. Vaccination with GS-4774 has been shown to induce HBV-specific T cells but lack the suppression HBsAg levels [51]. Similar results were obtained with TG-1050, which is based on an adenoviral system that encodes several HBV proteins [52].

Taken together, it is most likely that a “functional cure” will only be reached when the antiviral immune response against HBV is boosted via the activation of the innate and/or adaptive immune system. This immune activation in turn will only be achievable when HBsAg levels are suppressed by DAAs, as HBsAg may directly suppress the innate immune system [41].

## 3. Current Therapy of Chronic Hepatitis C

### 3.1. Indication for Antiviral Therapy

The therapeutic goal of antiviral therapy for chronic HCV infection is persistent virus suppression (SVR = “sustained virologic response”), which is defined by a lack of HCV RNA detection 12 weeks after the end of therapy. As the eradication of the virus does not lead to protective immunity, new infections are possible. Thus, a reinfection incidence of 6.4 per 100 patient years in patients with active intravenous drug use has been described [53]. Achieving an SVR is associated with a significant reduction in mortality, HCC development, and the need for a liver transplant [54,55]. While these positive effects are most obvious in patients with advanced fibrosis or compensated liver cirrhosis, they are less prevalent in patients with decompensated liver cirrhosis [56,57].

Every patient with a replicative HCV infection should be treated with antiviral therapy, provided that she or he will benefit from this treatment with respect to morbidity or mortality; when life expectancy is very limited, de novo therapy makes little, if any, sense, however. In the case of an initial diagnosis of HCV infection with the typical constellation of a chronic infection, antiviral therapy can be started immediately. Elevated transaminases and/or evidence of fibrosis are not necessary conditions. For patients with advanced fibrosis or cirrhosis, there is an urgent indication for treatment. Extrahepatic manifestations, professional reasons, the elimination of the risk of transmission, coinfections with HBV or HIV, and a patient’s desire for treatment are also indications for treatment.

### 3.2. Therapeutic Options for the Treatment of Chronic Hepatitis C Infection

For different groups of patients, several interferon-free therapy options are available on the basis of HCV geno- and subtype, possible previous therapies, the presence as well as stage of liver cirrhosis, and kidney function. When choosing among the therapy options, the effectiveness in achieving an SVR, possible side effects or contraindications, concomitant diseases, drug interactions, the duration of therapy, and, if applicable, economic factors must be taken into account [58].

HCV therapy should be carried out with an interferon-free therapy regimen. In the case of known or foreseeable ribavirin side effects, a ribavirin-free therapy should preferably be used. Patients coinfected with the hepatitis B virus or HIV can be treated in a similar manner to HCV monoinfected patients. It should be noted that DAA therapy can rarely lead to HBV reactivation, as HCV activates the innate immune system, which in turn can suppress HBV replication [42,59,60].

1. Pangenotypic Regimes in DAA-Naïve Patients

*a. Glecaprevir/pibrentasvir*.

*aa. DAA-naïve patients without cirrhosis.* In patients without cirrhosis, the administration of glecaprevir (GPR) and pibrentasvir (PBR) leads to high SVR rates (8 weeks: 98%; 12 weeks: 99%) independent of numerous predictors, including HCV genotype, HIV coinfection, and non-DAA-based prior therapy [61,62,63,64,65]. Therefore, for all patients without cirrhosis, therapy with GPR and PBR for 8 weeks is recommended. For patients with HCV genotype 3 infection, this only applies to therapy-naïve patients, while therapy-experienced patients should be treated for 16 weeks.

*ab. DAA-naïve patients with compensated cirrhosis.* In patients with compensated cirrhosis, 8 (97.7% [66]) or 12 weeks (99% [67]) of therapy also lead to high SVR rates, independent of predictive factors. Therefore, treatment with GPR and PBR for all treatment-naïve patients with compensated cirrhosis is recommended for 8 weeks. For the retreatment of patients with HCV genotype 1, 2, and 4–6 infection, the duration of therapy is 12 weeks, whilst it is 16 weeks for previously treated patients with HCV genotype 3 infection.

*b. Sofosbuvir/velpatasvir*.

A large phase 3 study was performed in patients with HCV genotype non-3 infection using velpatasvir (VEL) in combination with sofosbuvir (SOF) for 12 weeks regardless of previous therapy, the presence of compensated cirrhosis, and numerous other predictors. In this study, an SVR rate of 99% was achieved [68]. In another phase 3 study, patients with HCV genotype 2 and 3 infection were included. Here, a 12-week therapy with VEL/SOF also led to an SVR rate of 99% [69]. As in a phase 2 study on patients with HCV genotype 1 or 2 infection, significantly lower SVR rates (77–90%) were observed when therapy was given for only 8 weeks [70]; VEL/SOF should be given to patients with HCV genotype 1, 2, and 4–6 infection for 12 weeks regardless of previous therapy and the presence of compensated cirrhosis. In HCV-genotype-3-infected patients without cirrhosis, the administration of VEL/SOF is recommended for 12 weeks. In patients with compensated cirrhosis, ribavirin can be added (Table 4).

2. Genotype-Specific Regimens in DAA-Naïve Patients

*Sofosbuvir/ledipasvir.* The combination of sofosbuvir (SOF) with the NS5A inhibitor ledipasvir (LDV) was the first approved interferon-free, fixed coformulated regime. It is approved for the antiviral treatment of patients with HCV genotype 1, 4, or 6 with or without liver cirrhosis. The standard duration of therapy is 12 weeks and can be shortened to 8 weeks in therapy-naïve, noncirrhotic patients with HCV genotype 1 infection and a viral load of <6 million IU/mL HCV RNA. In patients with liver cirrhosis and/or negative predictors (e.g., failure of prior therapy, platelet counts of <75,000/nL), ribavirin can be added. In clinical practice, however, SOF/LDV is only rarely used due to the approval of pangenotypic regimens [58].

*Grazoprevir/elbasvir.* In this regimen, the NS3/4A protease inhibitor grazoprevir (GZR) is combined with the NS5A inhibitor elbasvir (EBR). It is licensed for the treatment of genotype 1 and 4 infections. Independent of the presence of compensated liver cirrhosis, the standard duration of therapy is 12 weeks. In patients infected with HCV genotype 1a, an extension of therapy to 16 weeks as well as the addition of ribavirin in patients with an initial viral load of over 800,000 IU/mL should be considered to reduce the risk of treatment failure. An extension of therapy to 16 weeks should also be considered in the presence of HCV genotype 4 with an initial HCV RNA of >800,000 IU/mL. Both SOF/LDV and GZR/EBR are only rarely used due to the approval of pangenotypic regimens, however [58].

3. Retherapy of DAA Failures

Patients that have failed therapy with an IFN-free DAA regimen should be retreated with a combination of voxilaprevir (VOX), VEL, and SOF for 12 weeks. This includes patients who have failed combination therapies consisting of SOF plus one NS3 protease inhibitor (e.g., simeprevir) or NS5A inhibitor (e.g., daclatasvir, ledipasvir, and velpatasvir) as well as nucleoside-free first-generation therapies (e.g., grazoprevir plus elbasvir or paritaprevir plus ombitasvir, with or without dasabuvir), each with or without the additional administration of ribavirin. This recommendation is based on two phase 3 studies that included patients with all HCV genotypes, various previous therapies, and patients with cirrhosis. The SVR rates ranged between 96% and 98% [71].

4. Treatment of Special Patient Populations

*a. Patients with decompensated cirrhosis.* Due to possible liver toxicity, NS3/4A protease inhibitors such as GZR, glecaprevir, and VOX are contraindicated in patients with decompensated cirrhosis. Consequently, therapy is limited to SOF in combination with NS5A inhibitors such as VEL and LDV in this population. The indication for antiviral therapy is given for all patients in whom a liver transplant can be avoided in the medium or long term, usually in patients with a MELD (model of end-stage liver disease) score of <20. In patients with a short-term need for a liver transplant, the indication for an antiviral therapy is much more difficult. The advantages of viral eradication before transplantation need to be weighed against serious potential side effects of the antiviral drugs in already very sick patients. Therefore, each case should be discussed with a liver transplantation center.

*b. Patients with renal insufficiency.* Patients with severe renal impairment (GFR < 30 mL/min or dialysis) should be treated in an equivalent manner to patients without renal insufficiency, with the following therapy options: GPR/PBR for 8, 12, or 16 weeks or GZR/EBR for HCV genotype 1 or 4 for 12 or 16 weeks. Here, previous therapies, comedications, and any comorbidities should be taken into account. Studies show high SVR rates of 98% across all HCV genotypes for GPR/PBR [64] and 99% in patients with HCV genotype 1 infection for GZR/EBR [72]. The additional administration of ribavirin should be avoided. Regimens including SOF should not be given in severe renal impairment.

## 4. Current Therapy of Chronic Hepatitis D

### 4.1. Indication for Antiviral Therapy

HDV/HBV-coinfected patients are at higher risk for the development of liver cirrhosis compared to HBV-monoinfected individuals [2,8,9,73]. Thus, about 20% of all cases of liver cirrhosis in HBsAg-positive patients are due to HDV coinfection, which is associated with significantly increased mortality [2,8,9]. HDV infection is also an independent risk factor for the development of hepatocellular carcinoma, with a relative risk of 1.3 in HBV/HDV- and 7.1 in HBV/HDV/HIV-coinfected patients compared to HBV-monoinfected patients [74]. Therefore, all HDV patients should be evaluated as possible candidates for antiviral therapy.

### 4.2. Therapeutic Options for the Treatment of Chronic Hepatitis D Infection

*Pegylated IFN.* PEG-IFN is approved for the treatment of hepatitis B and is also effective against HDV [75]. HDV RNA levels can be suppressed by up to 47% via standard IFN or PEG-IFN therapy. In the two large, controlled, and prospective HIDIT studies, the response rate at the end of therapy was 23–48%. Twenty-four weeks after the end of therapy, only about 25% of patients had negative HDV RNA [76,77]. During the long-term follow-up, however, about 50% of the patients had a late HDV RNA relapse [78]. Successful IFN therapy is associated with a more favorable long-term course, as the risk of developing clinical complications of liver cirrhosis was lower in these patients [11,79,80,81]. When a loss in HBsAg was achieved, a very favorable long-term course could be observed [79,82].

*Nucleoside/nucleotide analogues.* NUCs against HBV have no direct antiviral activity against HDV. There are negative studies for famciclovir [83], lamivudine [84], entecavir [85], and adefovir [76]. Likewise, TDF in combination with PEG-IFN showed no additional effect compared to PEG-IFN alone [77]. Nevertheless, it can be assumed that the therapy principles recommended for HBV monoinfection are also applicable for HDV/HBV coinfection. In the majority of cases, however, patients with hepatitis D have low HBV DNA levels [86,87], and will not benefit from HBV DNA suppression.

*Bulevirtide.* BLV is an entry inhibitor at the sodium taurocholate cotransporting polypeptide (NTCP) receptor, and has recently been approved for the treatment of HBV/HDV coinfection. HBV and HDV use NTCP as a receptor for virus entry [88]. BLV has been tested in several phase 2 studies. The results showed that monotherapy with this compound resulted in a dose-dependent decrease in HDV RNA levels; however, the studies also found evidence that combination therapy with PEG-IFN may be more effective than BLV monotherapy [89]. In a phase 2b dose-ranging study, patients received one dose of BLV of 2 mg, 5 mg, or 10 mg in combination with TDF for 24 weeks. One patient group was treated exclusively with TDF monotherapy. At the end of the therapy, 46%, 47%, and 77% of patients, respectively, had a drop in HDV RNA of more than 2 logs compared to 3% in TDF monotherapy. While ALT levels also dropped, BLV had no effect on HBsAg levels [90].

BLV/PEG-IFN combination therapy for 48 weeks was also studied [91]. The median drop in HDV RNA was greater in the BLV/PEG-IFN group (−4.81 and −5.59 log for the combinations with 2 mg and 5 mg of BLV, respectively) compared to the PEG-IFN or BLV group (−1.30 and −2.84). A total of 53.3% of patients in the BLV 2 mg/PEG-IFN group and 26.7% of patients in the BLV 5 mg/PEG-IFN group were HDV-RNA-negative 24 weeks after the end of therapy. Additional data from a study with 10 mg BLV/PEG-IFNα-2a or 10 mg BLV/TDF for 48 weeks [91] showed that 86.7% of patients in the BLV/PEG-IFN group had undetectable HDV RNA at the end of therapy compared to 40% in the BLV/TDF group. Only combination therapy with PEG-IFN led to a decrease in HBsAg levels, while monotherapy with bulevirtide had no such effect. After the cessation of antiviral therapy, a rebound of HDV RNA was found in the majority of patients.

### 4.3. Future Options in the Treatment of Chronic Hepatitis D

*Pegylated interferon λ.* Pegylated interferon λ (PEG-IFNλ) is a type III interferon that stimulates cell-mediated immune responses through type III IFN receptors. In HDV patients treated with PEG-IFNλ, it was found that it is better tolerated than PEG-IFNα [92]. An SVR, as defined by a drop in HDV RNA levels by 2 logs 24 weeks after the cessation of antiviral therapy, was found in 36% of patients [93]. Further studies investigating IFNλ in patients with chronic hepatitis D are currently under way.

*Lonafarnib.* Lonafarnib is an orally active prenylation inhibitor that has demonstrated antiviral activity against HDV. Initial studies showed a significantly greater drop in HDV RNA levels when compared to a placebo, while no effects on HBsAg levels were seen. In phase II studies, lonafarnib was tested as a monotherapy, in combination with ritonavir, or in combination with PEG-IFN. The results showed that a combination therapy of low-dose lonafarnib with ritonavir or PEG-IFN was superior to a high-dose lonafarnib monotherapy with regard to antiviral activity and tolerability [94]. The LOWR-HDV-2 study [95] studied a triple regimen of 50 mg lonafarnib with ritonavir and PEG-IFN. A total of 63% of the patients in this study achieved the composite end point of a decline in HDV RNA of ≥2 logs and the normalization of ALT.

*Nucleic acid polymers.* Nucleic acid polymers (NAPs) have demonstrated very promising results when used for the treatment of HBV/HDV coinfection. In a phase 2 study, 12 patients with chronic hepatitis D and compensated liver disease received REP-2139 as an intravenous infusion once weekly, and after 15 weeks PEG-IFN was added for an additional 15 weeks. This was followed by PEG-IFN monotherapy for 33 weeks. HBsAg levels dropped in all patients during therapy, and 5 of 12 patients were negative for HBsAg at the end of therapy as well as positive for anti-HBs [96]. HDV RNA remained negative 18 months after the end of therapy in seven patients, and five patients were HBsAg-negative. During the long-term follow up, 7 of 11 patients were still HDV-RNA-negative 3.5 years after therapy [97].

## 5. Conclusions

In general, therapy for chronic viral hepatitis has improved significantly in the last two decades. The introduction of DAAs has revolutionized the treatment of chronic hepatitis C, as almost every patients can be cured from this infection with few, if any, side effects. Therefore, it is highly unlikely that new therapeutic developments will be seen for this entity in the future. The situation in hepatitis B is more complex, as chronic infection and disease progression can be controlled in most patients via the use of antivirals. A “functional cure”, however, is achievable in only a minority of treated individuals with the currently licensed drugs. In this case, a number of promising new therapeutic approaches are currently under investigation that are aiming to reach this goal. Finally, chronic hepatitis D remains the most challenging type of chronic viral hepatitis. While a novel compound has recently been licensed for the treatment of this disease, the overall results to control this infection and prevent disease progression are still not satisfactory. Here, further therapeutic approaches are desperately needed in the future.

## Figures and Tables

**Table 1:** Risk of HBV reactivation in HBsAg-positive, anti-HBc-positive patients (modified from [18]).

High risk (>10%)	B-cell depletion, anthracyclins, corticosteroids (>4 w, >10 mg/d), cyclophosphamide, stem cell transplantation, high-dose chemotherapy, and TACE/resection in HCC patients
Moderate risk (1–10%)	TNF inhibitors, cytokine/integrin inhibitors, tyrosine kinase inhibitors, mTOR inhibitors, JAK1/2 inhibitors, DAA therapy for HCV infection, and corticosteroids (>4 w, <10 mg/d)
Low risk (<1%)	Azathioprine, 6-mercaptopurine, methotrexate, intra-articular steroids, and corticosteroids < 1 w

**Table 2:** Risk of HBV reactivation in HBsAg-negative, anti-HBc-positive patients (modified from [18]).

High risk (>10%)	B-cell depletion, stem cell transplantation, and TACE/resection in HCC patients
Moderate to low risk (<10%)	Anthracyclins, corticosteroids (>4 w, >10 mg/d), TNF inhibitors, cytokine/integrin inhibitors, tyrosine kinase inhibitors, mTOR inhibitors, JAK1/2 inhibitors, corticosteroids (>4 w, <10 mg/d), DAA therapy for HCV infection, sorafenib for HCC, azathioprine, 6-mercaptopurine methotrexate, and intra-articular steroids

**Table 3:** Novel therapeutic strategies against chronic HBV infection (modified from [45]).

Mechanism of Action	Example Compounds
*a. Directly Acting Antivirals (DAAs)*	
Entry inhibitors	Bulevirtide
Capsid assembly inhibitors	JNJ-6379
HBsAg secretion inhibitors	REP-2139, REP-2165
Polymerase inhibitors	
Small interfering RNA (siRNA)	JNJ-3989
Antisense oligonucleotides (AO)	
*b. Activation of Innate Immunity*	
TLR-8 agonists	GS-9688
*c. Activation of Adaptive Immunity*	
Checkpoint inhibitors	ASC22
Therapeutic vaccination	GS-4774, TG-1050

**Table 4:** Pangenotypic regimens for DAA-naive patients without decompensated cirrhosis or advanced renal failure (modified from [58]).

Therapeutic Regimen	Duration (Weeks)	Patients without Cirrhosis		Patients with Compensated Cirrhosis		
		TN/TE	GT3 and TE	TN	TE	GT3 and TE
GPR/PBR	8	x		x		
GPR/PBR	12				x	
GPR/PBR	16		x			x
VEL/SOF	12	x	x	x	x	x

## Data Availability

Not applicable.
